# Quantifying the genetic parameters of feed efficiency in juvenile Nile tilapia *Oreochromis niloticus*

**DOI:** 10.1186/s12863-018-0691-y

**Published:** 2018-11-16

**Authors:** Hugues de Verdal, Marc Vandeputte, Wagdy Mekkawy, Béatrice Chatain, John A. H. Benzie

**Affiliations:** 1 CIRAD, UMR116 ISEM, TA B-116/16, 73 rue Jean-François Breton, 34398 Montpellier Cedex 5, France; 2 Worldfish, Jalan Batu Maung, 11960 Bayan Lepas, Penang Malaysia; 3 GABI, INRA, AgroParisTech, Université Paris-Saclay, F-78350 Jouy-en-Josas, France; 4 Ifremer, UMR9190 MARBEC, Chemin de Maguelone, F-34250 Palavas-les-Flots, France; 5 Animal Production Department, Faculty of Agriculture, Ain Shams University, Hadaeq Shubra, Cairo, 11241 Egypt; 6 School Of Biological Earth and Environmental Sciences, University College Cork, Cork, Ireland

**Keywords:** Feed conversion ratio, Tilapia, Heritability, Genetic estimations, Correlations

## Abstract

**Background:**

Improving feed efficiency in fish is crucial at the economic, social and environmental levels with respect to developing a more sustainable aquaculture. The important contribution of genetic improvement to achieve this goal has been hampered by the lack of accurate basic information on the genetic parameters of feed efficiency in fish. We used video assessment of feed intake on individual fish reared in groups to estimate the genetic parameters of six growth traits, feed intake, feed conversion ratio (FCR) and residual feed intake in 40 pedigreed families of the GIFT strain of Nile tilapia, *Oreochromis niloticus*. Feed intake and growth were measured on juvenile fish (22.4 g mean body weight) during 13 consecutive meals, representing 7 days of measurements. We used these data to estimate the FCR response to different selection criteria to assess the potential of genetics as a means of increasing FCR in tilapia.

**Results:**

Our results demonstrate genetic control for FCR in tilapia, with a heritability estimate of 0.32 ± 0.11. Response to selection estimates showed FCR could be efficiently improved by selective breeding. Due to low genetic correlations, selection for growth traits would not improve FCR. However, weight loss at fasting has a high genetic correlation with FCR (0.80 ± 0.25) and a moderate heritability (0.23), and could be an easy to measure and efficient criterion to improve FCR by selective breeding in tilapia.

**Conclusion:**

At this age, FCR is genetically determined in Nile tilapia. A selective breeding program could be possible and could help enabling the development of a more sustainable aquaculture production.

## Background

A major issue confronting the world today is how to sustainably feed the world’s rising human population (predicted to attain 9.6 billion in 2050) with less space available for farming [1]. Key components of strategies to address this problem include the use of novel sources of food such as insects, greater access to underutilized farming systems such as aquaculture and, most importantly, improving the efficiency of existing farming systems [2].

Farmed fish species offer a particular opportunity in this regard. Aquaculture production has grown rapidly in the last 40 years and world production of farmed fish in 2012 was 74 million tons [3], similar to the global production of beef cattle [4]. These farmed fish used around six times less feed than beef cattle to produce the same amount of body mass [5, 6] Improved efficiency of fish production would provide even more benefit and enable further sustainable development of a still underutilized food production system. However, lack of adequate technology for recording feed efficiency in aquatic species and consequently, lack of information on its basic genetic parameters in fish is a key impediment to the implementation of selective breeding required to achieve this goal.

The single greatest cost in intensive fish farming systems is feed, ranging from 30 to 70% of the total production costs [7, 8]. Reducing feed consumption for a given productivity level is therefore key to achieve economic sustainability of fish farming [9]. At the environmental level, improved feed efficiency is expected to have strong positive impacts at different levels. First, through a reduction of the amount of resources used, including fish oil and fish meals, thus contributing to the preservation of marine ecosystems. A better feed efficiency would also reduce nutrient outputs (nitrogen, phosphorus) that can be detrimental to the environment [10]. Finally, reducing feed consumption would also reduce energy consumption and greenhouse gases emissions due to feed production [11]. From a social perspective, improving feed efficiency (FE) in animal production should lead to a reduction of the competition for raw materials between humans and animals, and increase the quantity of food for humans, particularly the poorest, enhancing their access to proteins and balanced nutrition.

Feed efficiency can be improved through husbandry, feed formulation and by selective breeding. For example, rearing systems and feeding regimes can be customized to promote more efficient feed use and reduce unnecessary movement and therefore energy expenditure [12–14]. Careful formulation of feeds can provide for more efficient digestion and utilization of feeds [15], and reduce the amounts of fish oils in diets [16].

Much of the historical gain on feed efficiency in livestock agricultural animals has been obtained indirectly through selection for growth rate [17]. However, in fish species, there is no clear results indicating an improvement of feed efficiency with a selective breeding programme on growth. Growth could contribute to improve feed efficiency, but all studies are not going in the same way [8, 18–22]. Heritability estimates of feed efficiency in fish are scarce, and lower than in livestock (0 to 0.11 [20, 23] vs typically 0.21 to 0.30 [24, 25]). This has been thought to reflect basic differences between (poikilotherm) fish and (homeotherm) livestock with different energy allocation strategies [20, 26].

While challenging to measure in terrestrial species, FE, or more specifically feed conversion ratio (FCR = Feed intake.body weight gain^− 1^, representing the quantity of fed consumed to produce one unit of biomass) is even more complicated to measure in fish. As fish are reared in large groups in water, it is not possible to collect uneaten feed and measure individual feed intake. Special feed labeled with X-ray dense markers has been the main method used to estimate feed intake in fish [27–29]. Although noninvasive and accurate for a specific meal, the main disadvantages of this method are the stress associated with X-raying, but also the long recovery time (days or weeks) before the next possible assessment. This results in a relatively low repeatability of daily feed intake (*r* = 0.09 to 0.32 [19, 27, 30, 31]) since fish are not eating the same amount of feed from one meal to another. This can however be overcome by multiple measurements over a long period of time [30, 31]. Video methods used to determine individual feed intake (FI) in groups of fish were assessed recently for tilapia by de Verdal et al. [32]. From this previous study, it appeared that feed intake measurements over 11 meals with two meals per day was necessary to achieve 95% repeatability. In the present study, five growth traits, feed intake, and feed efficiency traits were measured in pedigreed families of the GIFT strain of Nile tilapia, *Oreochromis niloticus.* We used these data to estimate genetic parameters as well as the expected correlated response to different selection criteria to assess the potential of genetics as a means of improving FCR in the second most farmed fish in the world.

## Methods

### Rearing of fish

The study was carried out on the GIFT (Genetically Improved Farmed Tilapia) strain of Nile tilapia [33] selected for growth using a rotational breeding scheme. The families were produced by natural spawning from December 2014 to December 2015 at the WorldFish Jitra Research station, Malaysia. The pedigree of each fish was registered for the genetic parameter estimations. The total pedigree file included 3383 fish from the 15 generations of GIFT (fish measured in the present study and their ascendants). The experiment was undertaken using four batches representing 40 families (8 families starting in June 2015; 8 families starting in November 2015; 12 families starting in February 2016; 12 families starting in April 2016).

After donation and transfer to the Penang WorldFish station, fish were reared until the fry reached approximately 10 g of body weight. After a week of quarantine in tanks, the beginning of the experiment consisted to place 30 fish per family in 2 distinct 100 L indoor tanks (120 cm length, 35 cm width and 24 cm depth) in a recirculating water system.

In total, 1200 fish were studied during this experiment. The average temperature was 28 °C ± 1 °C and the photoperiod 12 L: 12D. After anesthesia with clove oil, each fish was tagged in the dorsal muscle with two colored T-bar tags (Avery Dennison tags, 25 mm) using an Avery Dennison Mark III pistol Grip tool. Each fish in a tank was tagged with a unique colored T-bar tag to be able to identify each fish individually. Commercial feed with 34% of crude proteins, 5% of crude fat, 5% of crude fiber and 12% of moisture was used to feed the fish. A specific daily feed ration was used, calculated following the equation of Mélard, et al. [34]:

$$ \mathrm{DFR}=14.23\ast \mathrm{Mean}\ {\mathrm{body}\ \mathrm{weight}}^{-0.322} $$

where DFR is the daily food ration (% of body weight per day) and mean body weight was the average body weight of the 15 fish within each aquarium. Using this feed ration, fish were not underfed and no competition for feed was observed during the experiment. The use of this calculation was done because feeding the fish until apparent satiation can vary lot according to the observer, thus reducing repeatability of the measurements and increasing the tank effects. If a group of fish stopped to eat before the end of the daily feed ration, the uneaten pellets were removed from the aquarium.

Mortality was recorded daily and the feed ration changed accordingly. At the beginning of the refeeding period, a relative high mortality was observed (around 100 fish in total), probably because these fish were unable to get up after the stress of fasting.

### Experimental design and trait measurements

**Fig. 1:**
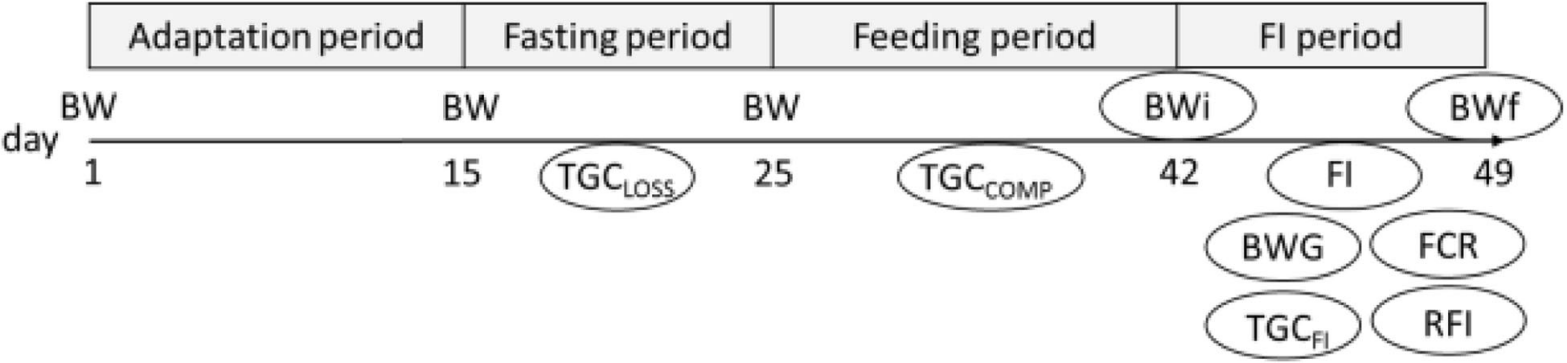
– Scheme of the different periods (days) designed in the experimental protocol and corresponding traits measured in each period (in circle). BW: body weight measurement; TGC_LOSS_: loss of weight during the fasting period estimated as thermal growth coefficient during the fasting period; TGC_COMP_: gain of weight during the feeding period estimated as the thermal growth coefficient during the refeeding period FI: feed intake measured for each fish reared in group; BWG: body weight gain during the FI period; TGC_FI_: thermal growth coefficient during the FI period; FCR: feed conversion ratio during the FI period; RFI: residual feed intake during the FI period

The experimental protocol was previously described in details by de Verdal et al. [32] and is summarized in Fig. 1. Body weight was measured at the beginning and end of the four time periods shown in Fig. 1: adaptation period (15 days), fasting period (10 days), feeding period (17 days) and FI period (7 days). After being tagged, fish were kept in groups in their aquarium to be acclimated to their new rearing system for 15 days before the beginning of the experiment as an adaptation period. Then, during the fasting period, fish did not have any feed and the aim was to measure the loss of weight during fasting. Following a fasting period, fish tends to compensate the loss of growth lived during the fasting period and by increasing their growth more than normally. It is known as the compensatory growth period, here noted as feeding period. Finally, after the growth compensation, feed intake was measured accurately using video records during the FI period.

The difference of weight between the beginning and end of each period of measurement was calculated as the thermal growth coefficient (TGC), which uses the cubic relation between BW and length to make growth rate linear over time and corrected for the water temperature (T) of the rearing environment during the measurement period:

$$ TGC=\left({BW}_2^{\left(1/3\right)}-{BW}_1^{\left(1/3\right)}\right)/\left(T\times \Delta  t\right)\times 100. $$

With *BW*_2_ the body weight at the end of the period; *BW*_1_ the body weight at the beginning of the period; T the rearing temperature and *∆t* the number of days of the measured period. The TGC is widely used in fish to be able to compare the growth of different fish species with different optimal rearing temperature. The loss of weight during the fasting period was noted as *LOSS*_*TGC*_ and the gain of weight during the compensatory refeeding period was noted as *COMP*_*TGC*_.

After the compensatory growth period, FI was recorded for each fish over a period of 7 days (13 meals) to estimate feed efficiency traits as detailed in de Verdal et al. [32]. During this individual FI period, feed was delivered to each aquarium pellet by pellet by hand through two pipes by an observer screened from view in order reduce the closeness between the person who give the pellets and the aquarium. This was done twice daily at 7.00 am and 1.00 pm. The first day, fish were weighed the morning and consequently, they received only one meal at 1.00 pm. Fish were fed until the calculated feed ration was finished. Video records of each meal was performed and video analyses were done to account for the number of pellets eaten by each individual fish during each meal. The day after the end of the FI measurement period, fish were anaesthetised with a high dose of clove oil and killed by decapitation. Fish were autopsied to measure different portions of the gastro-intestinal tract and sexed by visual observations of the gonads. The fish were too young to be reliably sexed using external morphology. Fish carcass were put in special bags, frozen and put in rendering wastes.

The Kinovea 0.8.15 software (Copyright © 2006–2011 – Joan Charmant & Contrib.) was used to analyse the videos of the meals. The main advantage of this software was to be able to play with the speed of reading and the zoom of the video for more accuracy. After weighing 500 pellets (mean = 16.4 ± SD = 1.76 mg, CV = 10.7%), the choice was done to consider that the pellet weight variability was low enough to assume that all the pellets had the same weight, which give the opportunity to calculate FI in grams. The total FI for an individual was calculated as the sum of the FI of all meals consumed. The thermal growth coefficient during the period when the feed intake was individually measured was noted as TGC_FI_. The body weight gain (BWG), during the feed intake measurement period was calculated as the difference in two body weight measurements taken at the beginning and end of the FI period.

The feed conversion ratio (FCR = FI/ BWG) was used as an indicator of feed efficiency.

The residual feed intake (RFI) was calculated as the difference between the feed consumed by a fish and the prediction of the feed consumption of this fish using a regression model estimation, taking into account the feed required for maintenance and growth [35]. The equation used to estimate RFI was as follows:

$$ \mathrm{RFI}=\mathrm{FI}-{\upbeta}_0-{\upbeta}_1\times {BW_f}^{0.8}-{\upbeta}_2\times \mathrm{BWG}\ \left(\mathrm{r}2\ \mathrm{of}\ \mathrm{the}\ \mathrm{model}=0.58\right) $$

with, β_0_, β_1_ and β_2_are the intercept of the regression, the partial regression coefficient of animal’s FI on metabolic body weight, and the partial regression coefficient of animal’s FI on BWG (measured as *BWG* = *BW*_*f*_ − *BW*_*i*_), respectively, BW^0.8^ is the metabolic body weight using 0.8 as the metabolic body coefficient, calculated by Lupatsch, et al. [36]. The more efficient fish are those with negative RFI, since these fish consume less than the average of fish with the same body weight and body weight gain whereas the less efficient fish are those with positive RFI, consuming more. The REG procedure of SAS (version 9.3, SAS Institute, Cary NC) was used to estimate the parameters of the regression equation.

### Estimation of genetic parameters

Genetic parameters were estimated by the REML (Restricted Maximum Likelihood) method using the VCE6 software [37, 38]. The following model was used for all the traits:

2 $$ {y}_{ijkl}=\mu +{Sex}_i+{Batch}_j+{Aquarium}_k+{Animal}_l+{e}_{ijkl} $$

Where *Sex*_*i*_ and *Batch*_*j*_ are fixed effects, *Aquarium*_*k*_ is a random environmental effect, and *Animal*_*l*_ is the random additive genetic effect of the animal l (*N* = 3383). The pedigree file included animals from the 15 generations of the selection process. Significance of fixed effects was tested using SAS (GLM procedure). As the aquarium effect was a random effect, it represented the common environmental effect, taking into account the non-genetic effect of the family as the fixed effect of the aquarium. Some of the studied traits showed very strong genetic correlations with each other, it was not possible to run a multiple trait analysis that include all traits, meaning that distinct bivariate analyses were performed. A total of 36 analyses were performed with two traits each. When the genetic parameter of a trait was estimated several times, the average of the estimation obtained was calculated.

To estimate the impact of the selection criterion on the other traits, the following equation from Falconer and Mackay [39] was used to compare the expected direct and indirect correlated response to selection (*CR*_*X*, *Y*_ with Y the selection objective and X the selection criterion) on the different criteria:

$$ {CR}_{X,Y}={i}_X\times \sqrt{\left({h}_X^2\times {h}_Y^2\right)}\times {rg}_{XY}\times {\sigma}_{P_Y} $$

where *CR*_*X*, *Y*_ is the expected correlated response of trait Y when selection is on X; *i*_*X*_is the intensity of selection on X, considered equal for all the traits and estimated equal to 2.34 for the Nile tilapia selection breeding program in the present study (corresponding to 2.85% of fish kept as breeders for the next generation, as was previously done in the GIFT Nile tilapia breeding program); ${h}_X^2$ and ${h}_Y^2$ are the heritability estimated for X and Y, respectively; *rg*_*XY*_ is the genetic correlation between X and Y; and ${\sigma}_{P_Y}$ is the standard deviation of Y phenotype. For the direct expected response, ${h}_X^2$ and ${h}_Y^2$ were similar (since X and Y were confounded) and *rg*_*XY*_ was equal to 1. Expected responses to selection were expressed in units of trait Y to improve.

## Results

### Phenotypic differences

Feed intake was measured on 40 families of the GIFT strain of Nile tilapia, as well as four growth characteristics (Fig. 1 for more details on the traits). Fish weighed on average 22.4 g and 31.9 g at the beginning and at the end of the FI period, respectively. This represent a growth of 9.33 g during the 7 days of FI measurement, representing a TGC of 0.16 during this period. During the same period, fish fed on average 8.35 ± 2.42 g. During fasting and refeeding periods, the TGC were respectively − 0.036 and 0.282 (with 24.9 and 17.5% of CVs, respectively).

Feed conversion ratio (FCR) was estimated on 953 fish. The mean FCR was 0.94 (SD = 0.21), and a large (22.1%) coefficient of variation (CV), close to that of body weight (Table 1). During the period of individual feed intake measurement, BWG was 10.3 g and 8.35 g for males and females, respectively, meaning that males grew 23.4% faster than females. Moreover, males consumed only 10.8% more feed than females during that period. In terms of FCR, males were significantly more efficient than females.

**Table 1:** Basic statistics (LS Means ± Standard Error) for all traits analysed

Trait^1^	N	Mean ± SD	Min	Max	CV (%)	LS Means ± SE	
						Males	Females
Growth traits and feed intake							
BWi	1029	22.4 ± 5.64	7.74	40.15	25.1	23.4 ± 0.17^a^	21.01 ± 0.18^b^
BWf	993	31.9 ± 8.26	10.04	56.61	25.9	33.7 ± 0.26^a^	29.4 ± 0.27^b^
TGC_LOSS_	997	− 0.036 ± 0.01	− 0.068	0.00	24.9	− 0.036 ± 0.001^a^	− 0.036 ± 0.001^a^
TGC_COMP_	995	0.282 ± 0.049	0.024	0.426	17.5	0.296 ± 0.002^a^	0.265 ± 0.002^b^
BWG	981	9.33 ± 3.14	0.76	18.3	33.7	10.3 ± 0.11^a^	8.35 ± 0.12^b^
TGC_FI_	993	0.16 ± 0.04	0.03	0.28	22.5	0.17 ± 0.001^a^	0.15 ± 0.002^b^
FI	949	8.35 ± 2.42	1.80	15.6	29.0	9.05 ± 0.09^a^	8.17 ± 0.09^b^
Feed efficiency traits							
FCR	953	0.94 ± 0.21	0.47	1.55	22.1	0.91 ± 0.01^a^	1.01 ± 0.01^b^
RFI	935	0 ± 1.53	−4.41	4.40	NR	−0.40 ± 0.06^a^	0.09 ± 0.07^b^

^1^BWi and BWf: BW at the beginning and at the end of the FI period (in g), respectively; *TGC*_*LOSS*_ Loss of weight during the fasting period estimated as thermal growth coefficient during the fasting period, *TGC*_*COMP*_ Gain of weight during the feeding period estimated as the thermal growth coefficient during the refeeding period, *BWG* Body weight gain during the FI period (g), *TGC*_*FI*_ Thermal growth coefficient during the FI period, *FI* Feed intake (g), *FCR* Feed conversion ratio (g.g^− 1^), *RFI* Residual feed intake (g)

NR CV are not relevant in these cases

^a-b^LS Means within row with different superscript are significantly different (P-value < 0.05)

The phenotypic correlations between all the measured traits are shown in Table 2 (below the diagonal). Growth traits, except TGC_LOSS_, were significantly and moderately to highly correlated with FI, with correlations ranged from 0.26 to 0.93.

**Table 2:** Estimates (± standard error) of heritability (highlighted in grey, on the diagonal), genetic correlations (above diagonal) and phenotypic correlations (below diagonal) for all the traits measured

Growth traits and feed intake								Feed efficiency traits	
Trait1	BWi	BWf	TGC_LOSS_	TGC_COMP_	BWG	TGC_FI_	FI	FCR	RFI
Growth traits and feed intake									
BWi	**0.65 ± 0.11**	**0.99 ± 0.01**	0.08 ± 0.26	ne	**0.86 ± 0.08**	0.33 ± 0.26	**0.77 ± 0.08**	0.11 ± 0.21	0.01 ± 0.18
BWf	0.95	**0.60 ± 0.11**	− 0.01 ± 0.25	ne	**0.93 ± 0.04**	0.47 ± 0.21	**0.77 ± 0.08**	0.08 ± 0.22	0.07 ± 0.19
TGC_LOSS_	0.03	0.02	0.23 ± 0.12	0.15 ± 0.29	− 0.18 ± 0.29	− 0.46 ± 0.41	0.46 ± 0.24	**0.80 ± 0.25**	**0.70 ± 0.22**
TGC_COMP_	0.80	0.80	0.07	**0.22 ± 0.06**	**0.70 ± 0.17**	**0.73 ± 0.26**	**0.55 ± 0.15**	0.18 ± 0.22	0.19 ± 0.23
WG	0.63	0.85	−0.02	0.64	**0.27 ± 0.08**	**0.75 ± 0.14**	**0.62 ± 0.13**	−0.07 ± 0.24	−0.14 ± 0.22
TGC_FI_	0.26	0.54	−0.03	0.41	0.88	0.10 ± 0.06	0.25 ± 0.26	−0.29 ± 0.28	−0.30 ± 0.25
FI	0.6	0.69	0.05	0.52	0.67	0.53	**0.45 ± 0.09**	**0.67 ± 0.15**	**0.63 ± 0.12**
Feed efficiency traits									
FCR	−0.11	− 0.25	0.09	− 0.19	−0.46	− 0.55	0.26	**0.32 ± 0.11**	**0.97 ± 0.03**
RFI	− 0.17	−0.18	0.09	−0.18	− 0.22	−0.19	0.46	0.83	**0.50 ± 0.10**

Bold indicates that the estimate significantly differs from zero

*ne* Non estimable due to a non-convergence of the estimation model

^1^BWi and BWf: BW at the beginning and at the end of the FI period, respectively; *TGCLOSS* Loss of weight during the fasting period estimated as thermal growth coefficient during the fasting period, *TGCCOMP* Gain of weight during the feeding period estimated as the thermal growth coefficient during the refeeding period, *BWG* Body weight gain during the FI period, *TGC*_*FI*_ Thermal growth coefficient during the FI period, *FI* Feed intake, *FCR* Feed conversion ratio, *RFI* Residual feed intake

Feed conversion ratio and RFI (the residual feed intake) were highly phenotypically correlated (rp = 0.83), and FCR showed moderate phenotypic correlations with BWG and TGC_FI_.

### Genetic parameters

The genetic parameters for growth, FI, FCR and RFI are shown in Table 2. Except TGC_FI_ and TGC_LOSS_, heritability estimates were significant and moderate to high, ranging between 0.22 ± 0.06 for TGC_COMP_ to 0.65 ± 0.11 for BWi. Feed intake, FCR and RFI showed significant moderate to high heritability, estimated at respectively 0.45 ± 0.09, 0.32 ± 0.11 and 0.50 ± 0.10.

Generally, the trend of genetic correlations (above the diagonal in Table 2) was consistent with phenotypic correlations. However, there were some exceptions. While the phenotypic correlations between BWG and FCR and RFI were negative and significant (− 0.46 and − 0.22 respectively), the genetic correlations between those traits were much lower and not significantly different from zero (from 0.07 to 0.33 in absolute values). On the other hand, there were low phenotypic correlations between FCR, RFI and TGC_LOSS_ (0.09), but genetic correlations were all high (0.80 and 0.70, respectively), making TGC_LOSS_ a promising indirect indicator criterion for selection on FE.

Growth traits, except TGC_COMP_ and TGC_FI_, and FI were highly and positively correlated, with correlations ranged from 0.55 to 0.77.

The genetic correlation between FCR and RFI was high (0.97 ± 0.03), suggesting that these two traits share the same genetic basis.

### Expected response to selection

The expected responses to direct selection and indirect strategies on body weight, growth, feed intake, FCR and RFI are shown in Table 3, and are expressed in unit of improved trait.

**Table 3:** Expected responses (expressed in unit of the trait improved) to direct selection (diagonal, in grey) or to indirect selection on body weight and growth variation (BWi, BWf, TGC_LOSS_, TGC_COMP_, BWG, TGC_FI_)^1^ and on feed intake and efficiency (FI, FCR, RFI)^2^ if one trait is used in the selective breeding program

Traits^2^	Selection objective								
	*Growth traits and feed intake*							*Feed efficiency traits*	
	BWi	BWf	TGC_LOSS_	TGC_COMP_	BWG	TGC_FI_	FI	FCR	RFI
Selection criterion^1^									
BWi	**7.21**	**9.81**	4.22e-04	.	**2.21**	**6.67e-3**	**1.99**	0.02	0.02
BWf	**6.70**	**9.34**	4.49e-05	.	**2.27**	**8.93e-3**	**1.88**	0.02	0.12
TGC_LOSS_	0.34	0.06	**4.19e-03**	3.46e-03	0.27	5.44e-3	0.70	0.10	0.82
TGC_COMP_	.	.	6.12e-04	**2.25e-02**	1.04	**8.41e-3**	0.81	0.02	0.22
BWG	3.89	5.83	8.10e-04	**1.74e-02**	**1.63**	**9.49e-3**	1.02	−0.01	−0.18
TGC_FI_	0.92	1.81	**3.28e-03**	**2.87e-02**	0.75	**7.79e-3**	0.25	−0.02	−0.23
FI	4.40	6.08	2.60e-03	**1.72e-02**	**1.28**	3.95e-3	**2.05**	0.13	1.00
FCR	0.53	0.51	**3.92e-03**	4.87e-03	−0.12	−4.02e-3	1.19	**0.15**	**1.34**
RFI	0.06	0.55	**4.27e-03**	6.39e-03	−0.31	−5.09e-3	1.4	**0.18**	**1.71**

Bolds indicate high (> 75%) expected responses, respectively

^1^As an example, select for FCR would improve FCR by 0.15 g.g^− 1^ at each generation of selection, but would reduce BWG by 0.12 g, whereas select for BWG would increase BWG by 1.63 g but will not improve FCR, as the expected response to selection is − 0.01 g.g^− 1^

^2^BWi and BWf: BW in g at the beginning and at the end of the FI period, respectively; *TGCLOSS* Loss of weight during the fasting period estimated as thermal growth coefficient during the fasting period, *TGCCOMP* Gain of weight during the feeding period estimated as the thermal growth coefficient during the refeeding period, *BWG* Body weight gain in g during the FI period, *TGC*_*FI*_ Thermal growth coefficient during the FI period, *FI* Feed intake in g, *FCR* Feed conversion ratio in g.g^− 1^, *RFI* Residual feed intake in g

Selection for body weight (BWi or BWf) would consistently improve all growth traits except TGC_LOSS_ (with a gain equal to 85.7 to 138.6% of that of a direct selection for the target growth trait). Selection for BW would also increase FI by 91.5 to 96.9% of the gain that could be obtained by direct selection for FI (representing around 2 g of increase for the period of 13 meals measured). Due to the low genetic correlations between body weight and FCR and RFI, and to the increase of FI and BWG in the same time period, selection for body weight would only moderately improve FCR (by 0.015 and 0.023 g.g^− 1^ for BWf and BWi, respectively, representing an improvement of FCR of 1.60 and 2.45% per generation, for a selection for BWf and BWi, respectively).

Selection for growth characteristics other than BW (BWG or TGC_FI_) would improve growth, as expected, but would not improve FCR.

Selection for FCR or RFI would improve FCR by 0.150 and 0.181 per generation, representing an improvement of FCR by 16.0 and 19.3% per generation of selection. Such selection would not influence body weight and BWG of the fish, and would moderately reduce TGC_FI_ (by 4.02e^− 3^ and 5.09e^− 3^, respectively, representing a decrease of TGC_FI_ of 2.5 to 3.2% per generation) and reduce FI (by 14.3 and 16.8% per generation).

Finally, it is important to highlight that selecting for TGC_LOSS_ would improve FCR by 10.9% per generation (representing 0.102 g.g^− 1^).

## Discussion

Until now, the very few studies in fish that estimated genetic parameters of individual variation of feed efficiency traits used the X-ray methodology. This method has a poor repeatability of daily feed intake between two records (*r* = 0.09 to 0.32 [27, 30, 31]), and is thus likely to be unreliable since fish show strong variation in FI from meal to meal and from day to day [40, 41]. This problem has been solved by using repeated measurements over a long period of time. Using data with 0.1 to 0.3 repeatability of feed intake between two records, Kause et al. [30] estimated an increase of the repeatability to 0.25 and 0.56, respectively, when three records were used and until 0.72 if six measurements were recorded and pooled. Conversely, we showed previously that accurate measurement of feed intake of individual fish reared in groups can be achieved using videos assuming appropriate care is taken in the feeding procedure and FI is measured for several consecutive days together with growth [32]. The main advantages of the video method compared to the X-ray method is that it is possible to follow individual feed intake of fish during consecutive meals without stressing the fish by handling or anesthesia. While the results of de Verdal et al. [32] need to be taken carefully because tilapia were juvenile (less than 50 g and around 3 months at the end of the experiment), it seemed possible to use this methodology to measure accurately feed intake of several consecutive meals. This allowed to estimate accurately FCR and consequently, to estimate with good repeatability, the genetic parameters of feed efficiency. However, we need to highlight that even if the method was accurate, it was used only on juvenile Nile tilapia (final BW = 32 g on average), over a short period of time and fish were reared in aquariums, not in ponds, there normal rearing system. Practically speaking, this method also has its drawbacks, i.e. the need to feed fish pellet by pellet and the time needed for video recording analyses.

### Heritability of feed efficiency

The present estimate of FCR heritability is much higher than the few published estimates from previous studies, which ranged from 0 to 0.07 in rainbow trout *Oncorhynchus mykiss* and European whitefish *Coregonus larvaretus* [19, 20, 31] vs. 0.32 ± 0.11 in the present study. FI was also more heritable in the present experiment (h^2^ = 0.45 ± 0.09) compared to published values for salmonids species in the literature, ranging from 0.07 to 0.29 [19, 20, 31, 42]. These differences could be partly explained by species differences or fish size differences, since they were not at the same stage of their growth curve, but may also reflect the more accurate methodology used in the present study to measure FI. Importantly, although higher than previous estimates in fish, the present results are in line with genetic parameters found in terrestrial livestock species such as chicken (h^2^_FCR_ = 0.21 ± 0.02, h^2^_RFI_ = 0.46 ± 0.06 [25]) and pigs (h^2^_FCR_ = 0.34 ± 0.05 and h^2^_RFI_ 0.26 ± 0.05 on average [24]). More accurate data from a range of fish species will however be required to settle the matter generally.

The demonstration of moderately to highly heritable FCR and RFI strongly suggests that a selective breeding program including these traits could efficiently improve feed efficiency. The high genetic correlations between FCR and RFI suggests these two traits are driven by the same genetic basis or represent the same trait, and that the improvement of one of these traits would improve the other one.

However, it is important to remind that fish studied in the present study were juvenile Nile tilapia. It could be interesting to try the same experiment on other fish species and at different ages to ensure that a selective breeding program will have an impact on all the rearing period rather than just at small range of fish size.

### Correlations of growth and feed efficiency traits

FCR is particularly difficult to measure directly on candidates in breeding programs. The present method, albeit accurate, requested the analysis of around 280 h of video recordings to evaluate 1004 fish. Thus, we also assessed different strategies for improving FCR given the correlations with other growth traits that are easier to measure and are already recorded in fish breeding programs. Using phenotypic information, several authors predicted that selection for growth in fish should be associated with an improvement of feed efficiency [8, 18, 21], as phenotypic correlation between feed efficiency and growth ranges from 0.6 to 0.9. In the present study, while the phenotypic correlations between BWG, TGC_FI_ and FCR were moderate (− 0.46 and − 0.55, respectively), the genetic correlation were low and not significantly different from zero (− 0.07 ± 0.24 and − 0.29 ± 0.28, respectively). Thus, we expect that contrary to the general thinking, selection for growth (BWG or TGC_FI_) would not induce a correlated response on FCR, and the same was true for RFI. Selecting for BWG would improve BWG by 1.63 g at each generation, a 17.5% improvement per generation, but would decrease FCR by 0.009 g.g^− 1^ at each generation (corresponding to an improvement of 0.96% of FCR at each generation). On the other hand, selecting tilapia for FCR would decrease FCR by 0.15 g.g^− 1^ at each generation of selection, corresponding to a 16% improvement of FCR at each generation, but would increase BWG by only 0.12 g (1.26%) at each generation. These results differ from previous results using the X-ray methodology reported by Kause, et al. [19] and Quinton, et al. [20] who estimated that selection for high growth would substantially improve FCR as a correlated response. These different results could be explained by the methodology used to measure FCR but also by the fish species as the present work was performed on tropical freshwater fish rather than previous works were done on saline seawater fish adapted to cold water temperature. From the statement that the basal metabolism is high on tropical fish than on cold water [43], it could be hypothesize that part of the feed consumed by a tropical fish is not going to the growth whereas in cold water fish species, almost all the FI is going through the growth, and consequently, the correlation between FCR and growth would be higher in cold water than in tropical water fish species.

Furthermore, the age of the fish could have an impact on these correlations between FCR and growth. More work is needed to clarify these results.

### Practical traits and strategies for selection of feed efficiency

Successful use of a trait in a selective breeding program requires the trait to be accurately measured. Although the approach of de Verdal et al. [32] used in this study permitted an accurate measurement of individual feed intake and thus of FCR on 40 families, the method was time consuming and impractical for application in industry selection programs. The generation time of Nile tilapia is around 1 year and the selection criterion would need to be measured on all the fish (usually 100 or more families) within 1 year to be useful. Even if one were able to imagine a more efficient scale up within an industrial process the cost of such an approach is likely to be prohibitive.

An alternative and powerful way to add FCR or RFI into a selective breeding program would be to find easily measurable traits highly correlated with FCR or RFI. It was previously shown that the loss of weight during fasting and the gain of weight during compensatory growth were heritable and phenotypically correlated with RFI in European sea bass and rainbow trout *Oncorhynchus mykiss* [27, 44]. Heritability estimates for those traits in the present study were comparable to the estimations previously obtained on sea bass by Grima et al. [44]. These authors also concluded that compensatory growth when refeeding after a fasting period was not a useful trait to use to improve FCR in sea bass. The present results from tilapia were similar, and in our case this result was explained by the low genetic correlations between TGC_COMP_ and FCR or RFI.

However, selecting fish for TGC_LOSS_ could have a high impact in terms of FCR improvement. The genetic correlation between FCR and the loss of weight during fasting (TGC_LOSS_) was high (0.80 ± 0.25). As TGC_LOSS_ is easy to measure in fish, since it is just growth measured during a period of fasting, it could be an efficient indirect selection criterion to improve FCR. Selecting Nile tilapia for high TGC_LOSS_ could improve FCR by 0.102 g.g^− 1^ (corresponding to 68.3% of a direct selection for FCR), representing an improvement of 10.9% of FCR at each generation of selection.

These results are surprising and not expected, as the previous reports estimating the correlations between the loss of weight during fasting and feed efficiency were in the opposite direction. Grima et al. [44] estimated that sea bass losing less weight during fasting were more efficient at converting feed into body mass. The contrasting results from sea bass and tilapia might be explained in three ways. Firstly, Grima et al. [44] measured a group of 50 fish rather than individual fish, and the correlation was a phenotypic correlation rather than a genetic correlation. At the phenotypic level, the present correlation between the loss of weight and FCR was not significantly different from zero. Secondly, sea bass lives in a relatively cold temperature and in seawater, while Nile Tilapia is a warm freshwater fish. It is possible that these two species do not store and use the same compartments (lipid, protein) in response to fasting. For example, for the same body weight, sea bass has much more perivisceral fat than tilapia and several authors have highlighted a relationship between feed efficiency and lipid percentage in cold water fish species *C. larvaretus* and *O. mykiss* [23, 31]*.* Thirdly, it is important to note that the present tilapia study focused on relatively small (around 30 g) and young fish (around 3 months) while those on sea bass were larger (50 g) and older (1 year to 18 months). It will be important to check for tilapia (or for any target species) if the genetic variation and correlations between traits are stable along time and the age at which selection is most effective. Recording individual FI and FCR is certainly easier in younger fish, but the impact of improving FCR on environment and economy is higher on larger fish. In this respect, if TGC_LOSS_ was also a good indirect selection trait for FCR in larger fish, it would be highly valuable as it remains easy to measure at any age.

## Conclusion

Our results demonstrated strong genetic control of FCR and RFI in juvenile Nile tilapia. Importantly, they demonstrated that at this age, improvements in FCR would not be achieved effectively by selection on growth alone in Nile tilapia. The estimates of response to selection under a number of selection strategies calculated from these data showed that FCR could be efficiently improved by direct selection or by indirect selection on RFI. However, both require tedious and time consuming measurement of individual feed intake. It is also important to highlight that fish studied here were juvenile. At the production level, feed is mainly consumed when fish are reaching harvest weight. Consequently, an estimation of the evolution of feed efficiency with the age of the tilapia could be helpful to understand at what age fish should be selected for feed efficiency improvement.

Interestingly, indirect selection for the relative weight loss at fasting would also yield substantial gains in FCR, and be much easier to implement. Additional work is required to measure FCR on older/larger fish and to estimate the impact of the fish age when the fasting period is performed. Furthermore, given the growing range and cost effectiveness of genomic tools, it could be particularly interesting to study the markers associated with FCR in view to use the genomic selection approaches to improve FCR. Our results show that selective breeding for feed efficiency in fish would be possible and would help enabling the development of a more sustainable aquaculture.

## Abbreviations

BW: Body weight
BWG: Body weight gain during the FI period
FCR: Feed conversion ratio during the FI period
FI: Feed intake measured for each fish reared in group
RFI: Residual feed intake during the FI period
TGC_COMP_: Gain of weight during the feeding period estimated as the thermal growth coefficient during the refeeding period
TGC_FI_: Thermal growth coefficient during the FI period
TGC_LOSS_: Loss of weight during the fasting period estimated as thermal growth coefficient during the fasting period
